# The impact of self-efficacy on health-related quality of life among community-dwelling older adults with chronic diseases: the mediating role of self-management behaviour

**DOI:** 10.3389/fpubh.2026.1875544

**Published:** 2026-07-30

**Authors:** Jihong Wei, Chengping Jian, Xiaoling Yan, Xiaomei Zhao, Zheng Xiao, Xiaoyu Dai

**Affiliations:** 1 Department of Hepatobiliary Pancreatic Spleen Surgery, Mianyang Central Hospital (Mianyang Hospital, Affiliated to the Medical School of University of Electronic Science and Technology), Mianyang, China; 2 Department of Ophthalmology, Mianyang Central Hospital (Mianyang Hospital, Affiliated to the Medical School of University of Electronic Science and Technology), Mianyang, China; 3 Department of Nursing, Jiange County People's Hospital, Guangyuan, China; 4 Information Center, Jiange County People's Hospital, Guangyuan, China; 5 MDT Platform, Mianyang Central Hospital,(Mianyang Hospital, Affiliated to the Medical School of University of Electronic Science and Technology), Mianyang, China; 6 Department of Nephrology, Mianyang Central Hospital, Mianyang, China; 7 President Office of Jiange County People’s Hospital, Guangyuan, China

**Keywords:** chronic disease, mediating effect, older adults, quality of life, self-efficacy, self-management behaviour

## Abstract

**Objective:**

To examine the relationships among self-efficacy, self-management behaviour and quality of life in community-dwelling older adults with chronic conditions, to test the mediating effect of self-management behaviour on the relationship between self-efficacy and quality of life, and to provide a theoretical basis for the development of a community-based chronic disease management nursing model led by specialist nurses.

**Methods:**

A convenience sampling method was used to recruit 189 older patients with chronic diseases from a community health service centre in Jiange County, Guangyuan City, Sichuan Province, between November 2025 and February 2026. Participants completed a general information questionnaire, the Chinese version of the Self-Efficacy for Managing Chronic Disease Scale, the Self-Management Behaviour Scale for Chronic Patients, and the Short Form 12 Health Survey. Data were analysed using SPSS 26.0, employing descriptive statistics, independent-samples t-tests, one-way analysis of variance, and Pearson correlation analysis. The mediating effect was examined using the PROCESS macro and verified with the bias-corrected bootstrap method.

**Results:**

Among community-dwelling older adults with chronic diseases, the mean score for the physical component summary (PCS) of HRQOL was 45.87 ± 8.92, and that for the mental component summary (MCS) was 48.23 ± 9.14. The mean score on the Chronic Disease Management Self-Efficacy Scale was 47.63 ± 12.84, and the mean self-management behaviour score was 72.41 ± 9.86. Pearson correlation analysis showed that SF-12 scores were positively correlated with C-SEMCD scores (*r* = 0.523, *p* < 0.01) and with self-management behaviour scores (*r* = 0.481, *p* < 0.01), and that C-SEMCD scores were positively correlated with self-management behaviour scores (*r* = 0.562, *p* < 0.01). Mediation analysis verified the partial mediating role of self-management behaviour. The indirect effect was 0.187 (38.6% of the total effect) for the PCS model, and the indirect effect was 0.203 (41.2% of the total effect) for the MCS model.

**Conclusion:**

Among community-dwelling older adults with chronic diseases, self-efficacy has a direct correlation with health-related HRQoL, and also correlates with HRQoL indirectly via self-management behaviour. Self-management behaviour acts as a partial mediator between the two variables. In clinical nursing practice, targeted efforts to improve patients’ self-efficacy may help foster positive self-management behaviours and may ultimately contribute to better HRQoL.

## 1 Introduction

As global population ageing accelerates, data indicate that non-communicable chronic diseases accounted for 74% of deaths worldwide in 2025 (1), marking humanity’s formal entry into the ‘chronic disease era’, with the overwhelming majority of chronic disease mortality and disease burden concentrated among people aged 70 years and over. According to World Health Organization projections, the global population aged 65 years and above is expected to reach 1.6 billion by 2050, representing more than 16% of the total population (2). By the end of 2025, China’s population aged 65 years and over numbered approximately 224 million, constituting 15.9% of the national population (3), and the prevalence of chronic diseases approached 80%. Among those aged 60 years or older, around 75% live with at least one chronic condition, and over 40% have multiple chronic conditions (multimorbidity) (4, 5). The high prevalence of disorders such as hypertension and diabetes, together with frequent multimorbidity, imposes a heavy burden on older adults’ health, family caregiving and social healthcare systems (6, 7). The protracted course of chronic diseases demands long-term management, and older patients—affected by physiological decline and low health literacy—experience severely impaired quality of life.

Quality of life is a core indicator for evaluating the effectiveness of chronic disease management. Health-related quality of life (HRQoL) is a specific subset of general quality of life. It focuses on individuals’ physical function, mental state and social functioning affected by diseases, which is distinctly different from overall quality of life that covers economic status, living environment and other non-health factors. The SF-12 scale adopted in this study is a mature tool designed to assess HRQoL. The protracted course of chronic diseases demands long-term management, and older patients—affected by physiological decline and low health literacy—experience severely impaired HRQoL (8). A recent systematic review confirmed that multimorbidity is the primary risk factor for reduced HRQoL in older adults populations, and increases the difficulty of daily disease management (7). Social cognitive theory posits a dynamic, reciprocal interaction among personal behaviours, cognitive factors and environmental influences. Self-efficacy, a central concept within this theory, refers to an individual’s belief in his or her capability to organise and execute specific courses of action; it has been confirmed as a key mediating variable in health behaviour change among patients with chronic conditions (9–11). Research has demonstrated that individuals with high self-efficacy tend to adopt positive health behaviours, which is accompanied by better disease control and higher HRQoL (12).

.Self-management behaviour encompasses the set of actions individuals undertake to manage disease symptoms, treatment regimens, and the physical, psychological and social consequences of chronic illness (13). In the context of chronic disease management, self-management behaviours include dietary management, exercise management, medication adherence, emotional management and symptom monitoring, amongst others (14, 15). Extant studies have predominantly focused on the direct relationship between self-efficacy and quality of life, with fewer investigations examining the mediating mechanism of self-management behaviour within this relationship (16). Furthermore, self-efficacy may influence health outcomes by shaping individuals’ behavioural choices and persistence; consequently, self-management behaviour may constitute a critical mediating variable linking self-efficacy to quality of life.

Although the direct relationship between self-efficacy and HRQoL has been well documented in various chronic disease populations, the pathways through which self-efficacy exerts its effects remain insufficiently explored, particularly among older adults with multimorbidity in community settings. Recent evidence suggests that self-management behaviour may act as a key behavioural mechanism linking self-efficacy to HRQoL (17). However, most existing studies have treated self-management behaviour as either an outcome or a predictor, rather than formally testing its mediating role in the self-efficacy–HRQoL relationship (18, 19).

Although prior research has explored the pairwise associations among the three constructs and has suggested a potential mediating role of self-management behaviour (20), the precise pathways of influence remain unclear and the mediating mechanism has not been examined in depth. To address this gap, Focusing on community-dwelling older adults with multimorbidity at the county level, this study quantitatively estimated and compared the distinct mediating roles of self-management behaviour on the Physical Component Summary (PCS) and the Mental Component Summary (MCS). It provides local empirical evidence for grassroots chronic disease management research in older adults and further extends the application of social cognitive theory within the context of chronic care nursing in China.

## 2 Methods

### 2.1 Study design and participants

A convenience sampling method was employed to recruit older adults patients with chronic diseases from a community health service centre in Jiange County, Guangyuan City, Sichuan Province, between November 2025 and February 2026. Jiange County is a typical county integrating rural and semi-urban areas in northern Sichuan. The local older adults population is mainly composed of rural residents and residents of county urban areas, with general characteristics such as relatively low average education level, dominant family pension mode, and high prevalence of hypertension, diabetes and other common chronic diseases. According to simulation studies by Fritz and MacKinnon (21), a sample size of approximately 150 is often recommended as a minimum to achieve adequate statistical power (e.g., 0.80) for detecting a mediation effect of medium size when using bias-corrected bootstrap methods. Inclusion criteria were: (a) age ≥ 60 years; (b) diagnosis of at least one chronic disease confirmed by a medical institution; (c) residence in the community for ≥ 6 months; (d) clear consciousness and basic communication ability; and (e) provision of informed consent and voluntary participation. Exclusion criteria were: (a) severe cognitive impairment or mental illness; (b) coexisting advanced malignant tumours or terminal organ failure; and (c) severe visual or hearing impairment preventing questionnaire completion.

### 2.2 Measures

#### 2.2.1 Demographic questionnaire

A general information questionnaire was used to collect data on age, sex, ethnicity, educational level, marital status, living arrangement, household monthly income per capita, type of health insurance, diagnosed chronic diseases, disease duration and presence of complications.

#### 2.2.2 Chinese version of the self-efficacy for managing chronic disease scale (C-SEMCD)

The Chinese version of the Self-Efficacy for Managing Chronic Disease Scale (C-SEMCD) was used to assess participants’ self-efficacy in chronic disease management. Originally developed by Lorig et al. (22) at Stanford University, the scale was later translated into Chinese by Wang et al. (23). It consists of 6 items, each scored on a 10-point Likert scale, with 1 representing “no confidence at all” and 10 representing “complete confidence.” Total possible scores range from 6 to 60, and higher scores reflect greater self-efficacy. In this study, the Cronbach’s *α* coefficient for the scale was 0.934.

#### 2.2.3 Self-management behaviour scale for chronic patients

The Self-Management Behaviour Scale for Chronic Patients, developed by Chinese scholar Qiao Han (24), was adopted to assess participants’ self-management practices. The scale encompasses 20 items across five domains: dietary behaviour, exercise regulation, medication compliance, emotional adjustment, and family and social support. All items are scored on a 5-point Likert scale, resulting in a total score ranging from 20 to 100, where higher scores correspond to more favourable self-management behaviours. The Cronbach’s *α* coefficient for this scale in the current study was 0.845.

#### 2.2.4 Short form 12 health survey (SF-12)

The 12-item Short Form Health Survey (SF-12) was used to evaluate participants’ health-related quality of life. Originally developed by Ware et al. (25), the scale has been culturally adapted and validated for use among Chinese populations by Lam et al. (26). The tool includes 12 items that measure two core components: the Physical Component Summary (PCS) and the Mental Component Summary (MCS). Raw scores are transformed into a standardised 0–100 scale using norm-based scoring algorithms, with higher scores reflecting superior quality of life. In this study, the Cronbach’s *α* coefficient for the SF-12 was 0.91.

### 2.3 Data collection

Trained researchers explained the study purpose and questionnaire instructions to potential participants. After obtaining informed consent, electronic questionnaires were administered anonymously. Participants accessed the survey via a QR code linked to Wenjuanxing, a Chinese online survey platform. For participants with limited literacy or poor eyesight, the researcher read each item aloud and assisted in completing the responses.

### 2.4 Data analysis

All analyses were conducted in SPSS version 26.0. Before parametric analyses, we tested the key statistical assumptions. Normality was checked via Shapiro–Wilk tests and graphical methods. Levene’s test was used for homogeneity of variances. Multicollinearity among variables was assessed by VIF, and regression model diagnostics including residual distribution and outliers were also conducted. All assumptions for parametric methods were met in the present study. Descriptive statistics were used to summarise participant characteristics: categorical variables are reported as frequencies and percentages, while normally distributed continuous variables are presented as means ± standard deviations (x̄± s). Group comparisons of self-efficacy, self-management behaviour, and quality of life across different participant subgroups were performed using independent-samples t-tests or one-way analysis of variance (ANOVA). Pearson correlation coefficients were calculated to explore bivariate associations between the three main study variables. Mediation analysis was carried out using Hayes’ PROCESS macro (version 3.5, Model 4). In this model, self-efficacy was specified as the independent variable (X), self-management behaviour as the mediator (M), and the physical and mental component summary scores of quality of life as separate dependent variables (Y), with demographic variables included as covariates. A bias-corrected bootstrap procedure with 5,000 resamples was applied to generate 95% confidence intervals for the indirect effects. The threshold for statistical significance was set at *p* < 0.05.

### 2.5 Ethical considerations

The study was approved by the Medical Ethics Committee of Jiange County People’s Hospital (ID: S20250283-02). Electronic informed consent was obtained at the commencement of the online questionnaire; participants were required to actively opt in. If a participant chose not to consent, the survey was automatically terminated and proceeded only upon agreement. All participants were informed of their right to withdraw from the study at any time without penalty.

## 3 Results

### 3.1 Demographic characteristics

A total of 202 questionnaires were distributed, and 189 valid responses were returned, yielding an effective response rate of 93.6%. Seven questionnaires were excluded due to >20% missing key variables, and six were excluded owing to patterned responses (e.g., selecting the same option for all items). Of the 189 older adults patients with chronic diseases, 92 (48.7%) were male and 97 (51.3%) female; the mean age was 67.84 ± 8.36, with a range of 60–91 years. Educational attainment was distributed as follows: primary school or below, 86 (45.5%); junior high school, 58 (30.7%); senior high school/technical secondary school, 32 (16.9%); and college or above, 13 (6.9%). Regarding marital status, 134 (70.9%) were married, 17 (9.0%) divorced or separated, and 38 (20.1%) widowed. Living arrangements comprised: living alone, 31 (16.4%); living with spouse, 108 (57.1%); and living with children, 50 (26.5%). Monthly household income per capita was < ¥2,000 for 63 participants (33.3%), ¥2,000–3,999 for 87 (46.0%), and ≥ ¥4,000 for 39 (20.6%). The types of health insurance comprised urban employee basic medical insurance (112, 59.3%) and urban and rural resident basic medical insurance (77, 40.7%). The prevalence of specific chronic conditions was as follows: hypertension, 142 (75.1%); diabetes, 78 (41.3%); coronary heart disease, 45 (23.8%); stroke, 23 (12.2%); chronic obstructive pulmonary disease, 19 (10.1%); and chronic kidney disease, 16 (8.5%). A total of 124 participants (65.6%) had two or more chronic conditions. Disease duration was < 5 years for 68 participants (36.0%), 5–10 years for 72 (38.1%), and > 10 years for 49 (25.9%). Complications were present in 41 participants (21.7%).

Older adults with chronic diseases had a mean total self-efficacy score of 47.63 ± 12.84 and a mean item score of 7.94 ± 2.14. The total self-management behaviour score was 72.41 ± 9.86, with a mean item score of 3.62 ± 0.49; dimension-specific scores are presented in Table 1. For HRQoL, the physical component summary (PCS) score was 45.87 ± 8.92, and the mental component summary (MCS) score was 48.23 ± 9.14.

**Table 1:** Scores for dimensions of self-management behaviour among older adult patients with chronic diseases (*n* = 189).

Dimension	Number of items	Dimension total score	Item mean score
Dietary habits	4	14.86 ± 2.57	3.72 ± 0.64
Exercise management	4	13.42 ± 3.18	3.36 ± 0.80
Medication adherence	4	15.21 ± 2.09	3.80 ± 0.52
Emotional management	4	13.95 ± 2.86	3.49 ± 0.72
Family and social support	4	14.97 ± 2.43	3.74 ± 0.61
Total self-management	20	72.41 ± 9.86	3.62 ± 0.49

### 3.2 Correlation analysis

Pearson correlation analysis (Table 2) showed that self-efficacy was positively correlated with self-management behaviour (*r* = 0.562, *p* < 0.01), as well as with PCS (*r* = 0.483, *p* < 0.01) and MCS (*r* = 0.512, *p* < 0.01). Self-management behaviour was positively correlated with PCS (*r* = 0.446, *p* < 0.01) and MCS (*r* = 0.498, *p* < 0.01).

**Table 2:** Correlation matrix of self-efficacy, self-management behaviour and quality of life among older adult patients with chronic diseases.

Variable	Self-efficacy	Self-management behaviour	QoL-PCS	QoL-MCS
Self-efficacy	1			
Self-management behaviour	0.562**	1		
QoL-PCS	0.483**	0.446**	1	
QoL-MCS	0.512**	0.498**	0.634**	1

**P < 0.01.

### 3.3 Mediating effect of self-management behaviour on the relationship between self-efficacy and health-related quality of life

The PROCESS macro (Model 4) was used to examine the mediating role of self-management behaviour in the self-efficacy–health-related quality of life relationship, controlling for age, sex, educational level, disease duration and complications.

### 3.4 Mediation model with PCS as the dependent variable

Regression analysis (Table 3) indicated that self-efficacy positively predicted self-management behaviour (*β* = 0.431, *p* < 0.001); both self-efficacy (*β* = 0.298, *p* < 0.001) and self-management behaviour (*β* = 0.434, *p* < 0.001) positively predicted PCS. After the mediator was introduced, the direct effect of self-efficacy on PCS remained significant (*β* = 0.298, *p* < 0.001).

**Table 3:** Regression analysis of the mediating effect of self-management behaviour on the relationship between self-efficacy and PCS.

Regression equation	Overall fit index	Regression coefficients	Coefficient of determination	F statistic	Standardized regression coefficient	
Outcome variable	Predictor	*R*	*R^2^*	*F*	*β*	*t*
Self-management	Self-efficacy	0.562	0.316	86.24***	0.431	9.29
QoL-PCS	Self-efficacy	0.543	0.295	38.91***	0.298	4.58
	Self-management				0.434	4.97

***P < 0.001. Demographic variables were controlled in all models.

Mediation analysis (Table 4) revealed that the indirect effect of self-efficacy on PCS through self-management behaviour was 0.187 (Boot SE = 0.042, 95% CI = 0.112 to 0.278), not containing zero, thus indicating a significant mediating effect. The direct effect was 0.298 (Boot SE = 0.065, 95% CI = 0.169 to 0.427), and the total effect was 0.485 (Boot SE = 0.072, 95% CI = 0.343 to 0.627). The mediating effect accounted for 38.6% of the total effect.

**Table 4:** Mediation effects of self-management behaviour between self-efficacy and health-related quality of life.

Effect type	Effect	Boot SE	Boot 95% CI	Relative effect proportion
Variable: PCS				
Total effect	0.485	0.072	0.343 ~ 0.627	100%
Direct effect	0.298	0.065	0.169 ~ 0.427	61.4%
Indirect effect	0.187	0.042	0.112 ~ 0.278	38.6%
Variable: MCS				
Total effect	0.492	0.076	0.342 ~ 0.642	100%
Direct effect	0.289	0.068	0.154 ~ 0.424	58.8%
Indirect effect	0.203	0.041	0.134 ~ 0.295	41.2%

### 3.5 Mediation model with MCS as the dependent variable

Regression analysis (Table 5) showed that self-efficacy positively predicted self-management behaviour (*β* = 0.431, *p* < 0.001); self-efficacy (*β* = 0.289, *p* < 0.001) and self-management behaviour (β = 0.471, *p* < 0.001) positively predicted MCS. After the mediator was introduced, the direct effect remained significant (*β* = 0.289, *p* < 0.001).

**Table 5:** Regression analysis of the mediating effect of self-management behaviour on the relationship between self-efficacy and MCS.

Regression equation	Overall fit index	Regression coefficients	Coefficient of determination	F statistic	Standardized regression coefficient	
Outcome variable	Predictor	*R*	*R^2^*	*F*	*β*	*t*
Self-management	Self-efficacy	0.562	0.316	86.24***	0.431	9.29
QoL-MCS	Self-efficacy	0.581	0.338	47.52***	0.289	4.25
	Self-management				0.471	5.63

***P < 0.001. Demographic variables were controlled in all models.

**Figure 1:**
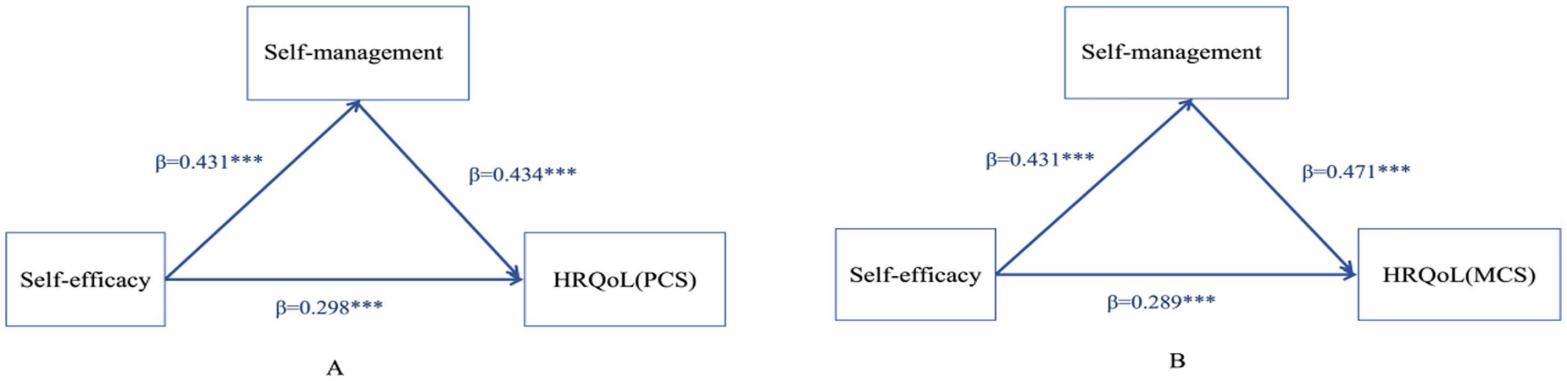
Path diagram of the mediating effect of self-management behaviour on the relationship between self-efficacy and health-related quality of life. **(A)** Mediating effect of self-management behaviour on the association between self-efficacy and health-related quality of life (PCS). Values represent standardized regression coefficients. ***p<0.001. **(B)** Mediating effect of self-management behaviour on the association between self-efficacy and mental component summary (MCS). Values represent standardized regression coefficients. ***p<0.001.

Mediation analysis (Table 4 and Figure 1) yielded an indirect effect of 0.203 (Boot SE = 0.041, 95% CI = 0.134 to 0.295), which was significant. The direct effect was 0.289 (Boot SE = 0.068, 95% CI = 0.154 to 0.424), and the total effect was 0.492 (Boot SE = 0.076, 95% CI = 0.342 to 0.642). The mediating effect accounted for 41.2% of the total effect.

## 4 Discussion

This study explored the relationships among self-efficacy, self-management behaviour, and HRQoL, and tested the mediating effect of self-management behaviour. The findings demonstrated significant positive correlations between self-efficacy and both self-management behaviour and the two dimensions of HRQoL. Moreover, self-management behaviour partially mediated the association between self-efficacy and both the physical and mental health components of HRQoL, with mediation proportions of 38.6 and 41.2%, respectively. These findings provide empirical evidence for understanding the mechanisms through which HRQoL may be associated with self-efficacy in older patients with chronic conditions, and offer theoretical guidance for clinical nursing interventions.

### 4.1 Relationships among self-efficacy, self-management behaviour and HRQoL

Pearson correlation analysis showed that self-efficacy was significantly positively correlated with self-management behaviour (*r* = 0.562, *p* < 0.01) and with PCS (*r* = 0.483, *p* < 0.01) and MCS (*r* = 0.512, *p* < 0.01). These results are consistent with previous domestic and international research. Cao et al. (27) identified a positive correlation between self-management and self-efficacy in post-PCI patients, with self-efficacy partially mediating the relationship between self-management and health-promoting behaviours. Amin et al. (28), in a study of community-dwelling older adults with hypertension, also demonstrated that self-efficacy was positively correlated with psychological well-being and played an important role in its promotion.

As Bandura articulated within social cognitive theory, self-efficacy beliefs do not operate in isolation (9),they interact with goal setting, outcome expectations and environmental factors to shape an individual’s motivation, behaviour and health outcomes. Recent research continues to support this framework. For example, a 2024 study focusing on a middle-aged population at risk found that self-efficacy, outcome expectations and environmental factors such as social support were significant predictors of physical activity and nutritional behaviours (29). Furthermore, a mediation analysis that explicitly incorporated goal setting and outcome expectations as mediating pathways demonstrated the indirect mechanisms through which self-efficacy beliefs influence behavioural change (30). These findings align with the central tenet of social cognitive theory that self-efficacy affects health outcomes partly through the motivational and regulatory mechanisms of goal setting and outcome expectations. Within the specific context of chronic disease management, older patients with higher self-efficacy are more inclined to initiate and sustain such actions. This positive behavioural pattern is correlated with better health outcomes and higher HRQoL in cross-sectional analyses (31, 32). The correlation results observed in the present study provide empirical support for this theoretical mechanism.

In our study, older patients with two or more chronic conditions accounted for 65.6% of the sample, a finding consistent with the global trend of rising multimorbidity rates among the ageing population. As reported in previous research (33), multimorbidity is closely associated with poorer HRQoL and more complex self-management requirements for older adults patients., owing to factors such as polypharmacy and the need to adhere to multiple lifestyle modifications. Self-management has been shown to improve patient adherence and subjective agency, thereby enhancing quality of life (34). Consequently, interventions aimed at strengthening self-efficacy to support complex self-management behaviours may hold particular value for older patients living with multimorbidity, with potential benefits for HRQoL.

### 4.2 Mediating role of self-management behaviour between self-efficacy and quality of life

The mediation analysis further clarified the associational pathway between self-efficacy and health-related HRQoL. In the PCS model, the indirect effect of self-management behaviour was 0.187, accounting for 38.6% of the total effect; in the MCS model, the indirect effect was 0.203, representing 41.2% of the total effect, both indicating significant partial mediation. The direct effect of self-efficacy on quality of life remained significant, highlighting that self-management behaviour serves as a partial mediator.

This finding suggests that the effect of self-efficacy on the HRQoL of older patients with chronic conditions is not entirely direct, but occurs partly through the mediating variable of self-management behaviour, supporting and extending the application of social cognitive theory within the chronic disease management domain (34). Specifically, Self-efficacy has a direct association with HRQoL, and also correlates with HRQoL indirectly through patients’ self-management behaviours. This is consistent with the chain mediating effect identified by Wang et al. (20) among Chinese COPD patients, wherein self-efficacy and self-management behaviours exerted a sequential mediating role between breathlessness beliefs and health-related quality of life, accounting for 28.91 and 33.01% of the total effect, respectively. Likewise, Zheng et al. (31) reported that self-efficacy significantly enhanced quality of life (*β* = −0.8557, *p* < 0.01), with self-management playing a moderated mediation role.

Notably, the mediating effect of self-management behaviour was proportionally larger in the MCS model (41.2%) than in the PCS model (38.6%), perhaps reflecting a more pronounced indirect influence on mental health. Some studies have indicated (28) that self-efficacy partially mediates the relationship between health behaviours and psychological well-being, with an indirect effect that can even exceed the direct effect. One possible reason is that the very process of enacting self-management behaviours inherently reinforces a sense of disease control and confirms self-worth; these psychological experiences act directly on mental health dimensions, strengthening patients’ sense of mastery and life satisfaction while alleviating negative emotions such as anxiety and depression (22, 35). By contrast, improvements in physical health typically require cumulative effects over a longer period and are directly influenced by biomedical factors such as disease severity and complications; thus, the mediating effect of self-efficacy through self-management behaviour on physical health is relatively smaller (34).

Additionally, the direct effect was significant in both models, suggesting that self-efficacy may be related to HRQoL via pathways independent of self-management behaviour. These may involve the regulation of psychological stress responses, an influence on treatment adherence, or an association with patients’ emotional states. Self-efficacy beliefs may be related to individuals’ affective reactions and stress levels, with those holding high self-efficacy reporting less anxiety and depression when facing disease-related challenges (36).

### 4.3 Limitations and future research

Several limitations should be acknowledged. First, the cross-sectional design precludes causal inference; longitudinal studies are needed to validate the mediation model. Second, the single-centre sample limits generalizability. Third, all variables were self-reported at a single time point. Although we implemented procedural remedies (anonymity, distinct scale anchors, separation of predictor and criterion variables) to minimize CMV, we acknowledge the absence of a post-hoc statistical test for common method bias as a limitation. Thus, the possibility that shared method variance may have influenced the observed associations, particularly the mediation proportions—cannot be entirely excluded. However, the differentiated correlation pattern (*r* = 0.446–0.562) and the distinct mediation proportions across PCS (38.6%) and MCS (41.2%) suggest that our findings are unlikely to be solely artifacts of CMV. Future research should incorporate objective clinical indicators and temporally separated measurements to more robustly address this issue.

### 4.4 Recommendations

Based on the results, we propose recommendations for community chronic disease management and clinical nursing. Grassroots health departments could consider including self-efficacy assessment in daily services, formulating targeted intervention programmes for rural and semi-urban older adults people, and conduct regular health education to promote self-management skills (37). Specialist nurses may consider hierarchical interventions: psychological encouragement and peer sharing help build confidence for patients with low self-efficacy, and all patients need guidance on standard diet, exercise and medication management (38). We also suggest integrating physical and mental care via emotional counselling and social support to enhance patients’ HRQoL (39), as well as designing personalized schemes for those with multimorbidity to ease the burden of self-management.

## 5 Conclusion

Self-efficacy, self-management behaviour and HRQoL were significantly positively correlated in this cross-sectional sample. Self-management behaviour partially mediated the relationship between self-efficacy and HRQoL, with mediation proportions of 38.6% for the physical health component and 41.2% for the mental health component. In nursing practice for older adults with chronic conditions, greater emphasis may be placed on assessing and enhancing self-efficacy to strengthen self-management behaviour, which may in turn be associated with better HRQoL.

## Data Availability

The raw data supporting the conclusions of this article will be made available by the authors, without undue reservation.
